# A Review of the Role of Neurotensin and Its Receptors in Colorectal Cancer

**DOI:** 10.1155/2017/6456257

**Published:** 2017-02-20

**Authors:** Shengyang Qiu, Gianluca Pellino, Francesca Fiorentino, Shahnawaz Rasheed, Ara Darzi, Paris Tekkis, Christos Kontovounisios

**Affiliations:** ^1^Department of Surgery and Cancer, Imperial College London, Chelsea & Westminster Hospital Campus, London, UK; ^2^Department of Colorectal Surgery, The Royal Marsden Hospital, Chelsea, London, UK

## Abstract

Neurotensin (NTS) is a physiologically occurring hormone which affects the function of the gastrointestinal (GI) tract. In recent years, NTS, acting through its cellular receptors (NTSR), has been implicated in the carcinogenesis of several cancers. In colorectal cancer (CRC), a significant body of evidence, from in vitro and in vivo studies, is available which elucidates the molecular biology of NTS/NTSR signalling and the resultant growth of CRC cells. There is growing clinical data from human studies which corroborate the role NTS/NTSR plays in the development of human CRC. Furthermore, blockade and modulation of the NTS/NTSR signalling pathways appears to reduce CRC growth in cell cultures and animal studies. Lastly, NTS/NTSR also shows potential of being utilised as a diagnostic biomarker for cancers as well as targets for functional imaging. We summarise the existing evidence and understanding of the role of NTS and its receptors in CRC.

## 1. Introduction

The hormone dependence of certain human cancers (e.g., breast and prostate cancers) is well described. As a result, hormonal modulation has become a cornerstone of therapy in these conditions. There is increasing recognition that cancers of the gastrointestinal (GI) tract, pancreas, and other organs express receptors for various endogenous host hormones. This raises the possibility of hormones' role in the proliferation of these cancers and therefore highlights the potential of these hormonal signalling pathways as targets for novel cancer diagnostic and therapeutic strategies. One of these promising candidates in GI cancers such as colorectal cancers (CRC) is the tridecapeptide neurotensin (NTS) [1–3].

NTS was first isolated in 1973 from the bovine hypothalamus and the digestive tract [4]. Its pharmacological and biochemical properties suggested its physiological functions are those of a neurotransmitter in the central nervous system (CNS) and a hormone peripherally. Centrally, it affects sensory and motor functions, temperature regulation, neuroendocrine control of the pituitary, and control of blood flow and blood pressure. In the gut, it is released by endocrine N cells in the jejunum and released after a meal, particularly those containing high lipid levels [5]. It has a range of paracrine and endocrine functions modulating vascular smooth muscle activity, gastrointestinal motility, and pancreaticobiliary secretions [6, 7]. Its action in the periphery is mediated by a G-protein-coupled receptor, neurotensin receptor 1 (NTSR1), whilst a second subtype, neurotensin receptor 2 (NTSR2), has mainly been identified in the central nervous system [8]. The third NTS receptor, NTSR3, is identical to sortilin, a 100 kDa protein with a single transmembrane domain [9]. Whilst NTSR1 and NTSR3 are found in a series of human cancer cell lines, the role of NTSR2 is much less studied in CRC [10].

NTS and its receptors have been implicated in the progression of a broad range of human cancers. These include cancers of the breast, prostate, lung, liver, and pancreas amongst others [11–15]. For example, Dupouy et al. reported that the upregulation of NTSR1 is associated with increased tumour size, number of metastatic lymph nodes, and Scarff-Bloom-Richardson grade of invasive ductal cell carcinomas of the breast [11]. Expression of NTSR1 was also increased in gastrointestinal stromal tumours [16]. The mechanism of action responsible for these effects is increasingly well understood, and NTS signalling has been shown to interact with multiple important oncogenic pathways [17]. Concurrently, there is increasing evidence of the roles NTS play in CRC.

This review consolidates the current evidence for the role NTS and its receptors play in the oncogenesis of CRC and identifies areas of translational research required to allow NTS to be used in the diagnosis and treatment of this prevalent cancer.

## 2. Material and Method

A search of the original published work was done by use of PUBMED, MEDLINE, and EMBASE databases. The following search terms used were “colorectal neoplasms” [MeSH Terms] OR colorectal cancer [Text Word] and (“neurotensin” or “neurotensin receptor”). Lists of references were obtained, and potentially relevant papers were retrieved. Reference lists in every paper were scrutinised to identify other possible relevant studies. All studies relevant to CRC were included.

## 3. Results

### 3.1. Neurotensin Signalling Pathways in Colorectal Cancer Cells

**Figure 1:**
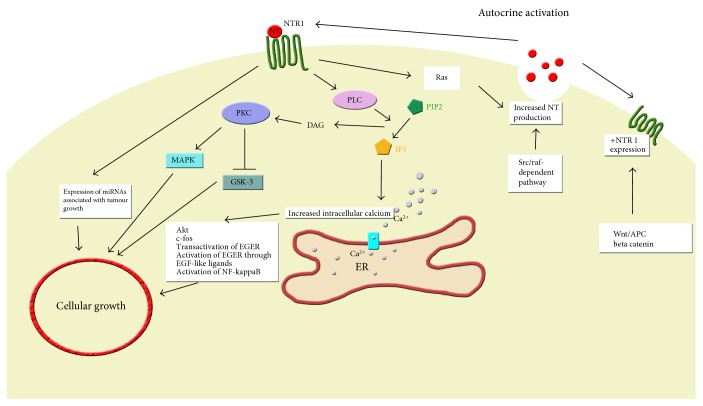
Neurotensin signalling in colorectal cancer cells. NTS: neurotensin, NTSR1: neurotensin receptor 1, PLC: protein lipase C, PKC: protein kinase C, MAPK: mitogen-activated protein kinase, GSK-3: glycogen synthase kinase-3, DAG: diacylglycerol, PIP2: phosphatidylinositol 4,5-bisphosphate, IP3: inositol trisphosphate, ER: endoplasmic reticulum, EGFR: epidermal growth factor receptor, APC: adenomatous polyposis coli.

Whilst prohormone convertase 1 has been implicated in the activation of NTS from its precursors in the GI tract, NTS appears to be derived from its precursor proneurotensin/neuromedian N via prohormone convertase 5 in CRC cell lines [18, 19]. Some colonic tumours synthesise and release NTS, resulting in autocrine control and cellular proliferation [20]. In cell cultures, physiological levels of NTS appear to stimulate the growth of many human colon cancer cell lines (SW480, SW620, HT29, HCT116, and Cl.19A) expressing NTSR1 [21]. The signalling pathways identified in NTSR1 are summarised in Figure 1.

Although NTSR1 was not normally detectable on human colonic epithelial cells, it appeared to be expressed as ectopic receptors in human colon cancer cells. Concurrently, neurotensin receptor-binding proteins and mRNA were undetectable on normal epithelial cells of the human colon. NTS receptors were found in 40% of human colon cancer cell lines in culture [22]. Moreover, NTSR1 expression on colon cancer cells appears to be upregulated by the Wnt/APC (adenomatous polyposis coli) signalling pathway, a well-known carcinogenesis pathway in CRC [23]. This may result in CRC cells which not only produce NTS, but also have increased sensitivity to its oncogenic effects, and contribute to their escape from the normal cell cycle.

In inflammatory bowel disease- (IBD-) related colon cancers, NTSR1 appears to be a *β*-catenin inducible gene. Precancerous and cancerous colonic lesions coexpressed NTSR1 and *β*-catenin, in the absence of NTS. Therefore, NTSR1 overexpression, during IBD-related oncogenesis at least, may be associated with an activation of the APC/*β*-catenin pathway [24].

With regard to downstream signalling, NTS was found to have little effect on cyclic nucleotide (cAMP and cGMP) levels in HT29 colonic cancer cell lines but strongly stimulates phosphatidylinositol turnover [25]. In one study, NTS stimulated inositol trisphosphate-mediated calcium mobilisation but not protein kinase C (PKC) activation in HT29 cells [26]. NTS receptors in HT29 cells appear to be coupled to phospholipase C (PLC). Activation of PLC leads to an increase in inositol phosphate levels, and this in turn resulted in Ca^2+^ release [26]. Calcium signalling is a key regulator of processes important in differentiation in CRC. Chowdhury et al. found that differentiation of HT29 colon cancer cells is associated with a remodelling of NTS-mediated Ca^2+^ signalling, a key stage in CRC cell transformation [27]. Confirmatory studies demonstrated that addition of NTS to human colon cancer cell lines resulted in calcium mobilisation as well as activation of the mitogen-activated extracellular signal-regulated kinase (MAPK/ERK) pathway and induction of c-fos expression [28–30].

NTS also has been shown to induce the phosphorylation of glycogen synthase kinase- (GSK-) 3 in HCT116 human colon cancer cell line via protein kinase C (PKC) [31]. GSK-3 is a regulator of a diverse range of cellular processes including cell growth. In HT29 cells, NTS induced DNA synthesis through phosphorylation of ERK and Akt via transactivation of epidermal growth factor receptors (EGFR). Similarly, in the HCT116 cell line, both PKC and EGFR pathways are implicated. In other cancers, NTS induces the autocrine activation of EGFR mediated through EGF “like” ligands [32]. However, Massa et al. found that, in HT29 and HCT116 cell lines, NTS-stimulated MAP kinase phosphorylation did not appear to involve EGFR and blocking EGFR alone may not be able to inhibit NTS-induced cancer proliferation in these cell lines [33]. These mechanisms of activation and transactivation need to be investigated further in CRC.

Further downstream, NTS was found to stimulate differential expression of 38 microRNAs, including miR-21 and miR-155, which have been associated with tumour growth and include nuclear factor kappa-light-chain-enhancer of activated B cells- (NF-*κ*B-) binding sites. NTS expression increased colony formation by HCT116 cells [34]. The NF-*κ*B pathways have also been implicated in NTS-induced inflammation and mitogenesis in colonocytes [35].

In addition to the activation of potentially oncogenic pathways in CRC cells, NTS production is increased within some CRC cells, resulting in autocrine control of cellular growth and proliferation. NTS promoter activity is increased by Src in Caco-2 human colon cancer cells, partly through a proximal AP-1/CRE promoter element. Additionally, Src regulation of the NTS promoter appears to be mediated through a Raf-dependent pathway [36]. Moreover, Ras, downstream of the NTSR1 signalling cascade, also targets the NTS promoter region resulting in increased NTS release [37, 38].

Clearly, the effects of NTS/NTSR1 signalling involve multiple signalling pathways and are cell line-dependent. Although many intermediary steps need to be clarified, the common end-effect of these pathways is colon cancer cell growth and proliferation [39].

Whereas the mitogenic effects of NTS were mediated through NTSR1, the role of the third NTS receptor, NTSR3, is less clearly defined. NTSR3 is not a G-protein-coupled receptor but appears to be a sortilin receptor. They were found not only predominantly in endoplasmic reticulum-Golgi compartments but also in the cell membrane of colonic cancer cells [40]. NTSR1 and NTSR3 exist as a heterodimer on the cell surface of HT29 human colon cancer cells. Upon stimulation with NTS, the receptor complex is internalised. This heterodimeric assembly appeared to modify the intracellular response to NTS [41]. Indeed, two forms of NTSR3 have been found on the HT29 cell line, a high molecular weight, membrane-associated form responsible for NTS endocytosis from the cell surface, and a lower molecular weight, intracellular form responsible for the sorting of internalised NTS to the trans-Golgi network, to its onward destinations, for example, the cell nuclei to carry out its mitogenic effects [42].

### 3.2. The Presence of Neurotensin in Colorectal Cancers In Vivo

Ulich et al. first described large numbers of neuroendocrine cells within well-differentiated colonic adenocarcinoma [43]. NTS mRNA, peptide, and receptor were found in resected human colon cancer specimens as well as in 4 well-known human colon cancer cell lines in vitro. In surgical specimens where NTS was identified in cancer cells, none was identified in adjacent normal bowel mucosa [20]. This suggested that the CRC cells had developed the propensity for NTS expression. The role of NTS in CRC was further highlighted by its detection in 13 human colon cancer cell lines and confirmation of its presence in numerous other colorectal cancer tissue specimens [18]. At the same time, NTSR expression in stromal versus epithelial cells was 35% and 12%, respectively, in CRC [44]. In a study of 30 patients with inflammatory bowel disease-related large bowel adenocarcinomas, dysplasias, and inflammation, the percentage of NTSR1-positive epithelial cells progressively increased from the inflammatory condition to adenocarcinoma and was significantly higher in adenocarcinomas than in inflammation [24]. Based on this evidence, it is possible that although NTS and NTSR may not play an active role in every case of colorectal cancer, it appears to play an important role in the growth of the cancer in which they are present. This theory is further supported by the work of Gui et al. who measured NTSR1 mRNA expression in normal colonic mucosa, adenomas, and colonic adenocarcinoma tissue specimens. Whilst NTSR1 mRNA expression was undetectable in differentiated epithelial cells of normal colonic epithelium, it was expressed at a moderate level in adenomas and adenocarcinomas. Higher level of expression was seen in adenocarcinomas' infiltrating margins. Tissue from lymphovascular invasion showed even higher intensity of expression of NTSR1 than the rest of the tumour. This further supports that increased NTSR1 expression may be an early event during colonic tumourigenesis and also contributes to tumour progression and aggressive behaviour in colonic adenocarcinomas [45].

Exogenous NTS causes significant proliferation of normal small bowel mucosa. In the colon, high doses and duration of NTS exposure (300 milligrams kg^−1^ administered three times daily for 10 days) stimulate the proliferation of colonic mucosa in rat models. Whereas there was an increase in cell volume in adult rats, the cell number was drastically increased in younger subjects [20]. NTS, at high doses, appeared to be able to stimulate cellular division and growth. NTS was found to accelerate colonic cancer carcinogenesis in animals. Rats injected with a colonic cancer carcinogen azoxymethane and NTS (200 micrograms kg^−1^ every other day for 40 weeks) significantly increased the number, size, and invasiveness of colon tumours [46]. Administration of NTS alone (300 and 600 micrograms kg^−1^) significantly stimulated growth in both murine colon tumours as well as human colon cancers xenografted into mice. Administration of NTS also resulted in significantly decreased survival of mice with CRC compared with the control group given saline injections [47]. A study compared sporadic and inflammation-induced colon cancers in which mice were exposed to either the carcinogens azoxymethane or azoxymethane with dextran sulfate sodium. NTSR1 stimulation appeared to significantly increase the development of sporadic cancers although not inflammatory cancers. Cancer rates were also significantly reduced in NTSR1-deficient mice compared to wild-type mice [48].

Growth of colon cancer cell lines xenografted into athymic mice was stimulated by NTS whilst the NTS receptor antagonist SR 48692 inhibited tumour growth [21]. Similarly, intraperitoneal administration of NTS increased the growth rate of HCT116 xenograft tumours in mice. Blocking miR-21 and/or miR-155 appeared to slow tumour growth [34]. Therefore, NTS/NTSR1 may be a potential target for preventive or therapeutic strategies in colon cancer. Kamimae et al. found that epigenetic silencing of NTSR1 was associated with reduction of invasive growth of colorectal tumours. They found that whereas noninvasive colorectal tumours tended to express high levels of hypermethylated NTSR1, invasive CRC tended to have unmethylated NTSR1. It is possible that methylation of specific CpG islands of the NTSR1 gene may result in gene silencing in CRC cells. The authors validated these findings against data sets from The Cancer Genome Atlas and observed an inverse relationship between the methylation levels on multiple probe sets of an Infinium BeadChip and levels of NTSR1 expression in CRC tissue [49]. However, the impact of methylation on any gene is dependent on the CpG islands studied. Therefore, more evidence is required to substantiate the expression or suppression of NTSR1 and NTS by methylation before it can be used as a reliable clinical prognostic marker.

The human tissue studies as well as the animal models above demonstrated, in observational studies at least, that NTS plays a role in carcinogenesis in some CRC subtypes. When it is involved, it played a significant role in the growth and aggressiveness of the cancer, affecting the survival outcome of subjects. Moreover, blockade of NTSR signalling attenuated CRC growth. This evidence raises the possibility for NTS and NTSR to be used as prognostic indicators in CRC, similar to the established use of oestrogen (ER) and Herceptin (HER) receptors in breast cancers. Like ER and HER receptors, this evidence also raises the tantalising possibility for NTSR to be a target for cancer therapy.

### 3.3. Clinical Applications of Neurotensin in Colorectal Cancer

Several groups have explored the potential use of the NTS pathway as target for functional imaging for the detection of cancers [50–53]. Furthermore, a fluorescence-based yeast biosensor has been developed that can monitor the activation of human NTSR1 by its agonist [54].

In a study evaluating blood NTS levels in colorectal cancer using 56 colorectal cancer patients and 15 controls, blood NTS and IL-8 levels differed between healthy and colorectal cancer patients. NTS values appeared to differentiate the control group from the cancer group. The value of plasma *NTS* ≥ 54.47 *pg*/*ml* at enrollment demonstrated a sensitivity of 77% and specificity of 90%, in predicting colorectal cancer confirmed by colonoscopy. This raises the possibility of using NTS as a diagnostic biomarker for colorectal cancer. Larger prospective studies are awaited [55].

### 3.4. Role of Neurotensin in Colorectal Cancer Treatment

Treatments which target the NTS/NTSR signalling pathway have been tested in cell culture and animal models. Sodium butyrate (NaBT), a potent histone deacetylase inhibitor (HDACi), dramatically decreased endogenous NTSR1 mRNA, protein, and NTSR1 promoter activity. HDACis are known to induce growth arrest, differentiation, and apoptosis of CRC cells. Therefore, the inhibitory effects of HDACi on CRC cells may in part be due to the suppression of the NTS/NTSR signalling pathway [56].

More directly, Iwase et al. found that NTS receptor antagonist SR48692 abolished the stimulatory effect of exogenous NTS administration on tumour growth in mice xenografted with human colon cancers. Interestingly, this is despite the lack of expression of NTSR in either tumour cells or xenografted tumours. They hypothesised that the trophic effect of NTS may be an indirect one [57].

Levy et al. treated both cultured human colon cancer cell line and mice colon cancers with combination of NTS and vasoactive intestinal peptide antagonists. The antagonist effectively inhibited cell growth in culture at nanomolar concentrations. Furthermore, colonic cancers harvested from mice treated with the antagonist showed reduced tumour volume, staging, lymphocyte infiltrate, and number of dysplastic crypts [58].

Other isolated studies have reported on the effect of dietary supplements and antioxidants on the effect of NTS on CRC cells. The natural dietary product, curcumin, appeared to inhibit NTS-mediated IL-8 protein secretion and colon cancer cell migration in culture [59]. Briviba et al. show that NTS and epidermal growth factor (EGF) caused a strong rise in the intracellular Ca^2+^ concentration, induced phosphorylation of ERK1 and ERK2, and stimulated growth of human carcinoma cells. The dietary antioxidant cyanidin found in fruits and vegetables appeared to inhibit the NTS- and EGF-induced cancer cell metabolism [29].

Another area of investigation is the use of NTSR1 overexpression in cancer cells as the target to deliver therapeutic molecules. This approach is made possible by NTSR1 endocytosis which resulted in delivery of molecules of interest inside the targeted cell. An example trialed in CRC cells was the targeted delivery of liposomes filled with doxorubicin and functionalised on the external surface with a branched moiety containing four copies of the 8–13 neurotensin (8–13 NTS) peptide. It dramatically increased the cytotoxic effects of this nanotherapeutic agent on HT29 cell lines [60]. Hernandez et al. and Hernandez-Chan et al. developed a NTS-based polyplex gene nanocarrier which has the potential nanomedicine-based application in the treatment of cancers which express NTSRs as well as diseases of the CNS-like Parkinson's disease [61, 62]. The prospect of NTS-based nanomedicine is an exciting one and awaits validation and translation into the clinical setting.

## 4. Discussion

Similar to other cancers, there is a convincing, albeit predominantly experimental, body of evidence to implicate NTS and its receptors in the carcinogenesis of human CRC. Human research on this subject remains sparse and lacking in strength. To date, only retrospective series have been reported in colorectal cancers. Prospective studies have been carried on in other cancers, for example, glioma, which demonstrated that increased NTS and NTSR1 expression is associated with significantly decreased 3-year survival [63]. The potential for NTS/NTSR pathway as novel targets for cancer therapies has been advocated in CRC as well as other cancers such as non-small-cell lung cancers [64]. NTS as a diagnostic and therapeutic target is being explored in numerous other cancers, for example, cancers in the pancreas, prostate, and breast [65]. In breast cancer, NTSR1-induced activation of EGFR and HER2 receptors rendered these cancers aggressive, yet highly responsive to lapatinib and metformin in mice [66]. However, translational studies into clinical settings are awaited in all of these cancers.

NTS and NTSR expression appear to be related to increased aggressiveness and invasiveness of colon cancers highlighting its potential role as a prognostic marker in CRC. Furthermore, NTS and NTSR can be easily quantified in resection specimens as a part of routine histopathological diagnosis. The NTS/NTSR status of a cancer, much like Herceptin and oestrogen receptor status in breast carcinoma, could be used to stratify patients for adjuvant chemotherapy agents. However, there has only been small retrospective case series reported thus far. The validity of NTS/NTSR as a prognostic factor requires improved understanding of the prevalence of human CRC which expresses them at a genetic and protein level. Cancer genetic databases may hold the answer. Prospective cohort studies are required to establish the effect of NTS or NTSR expression on disease aggressiveness, recurrence, and survival.

Functional imaging targeting NTS/NTSR is in the experimental stages but is fast approaching clinical validation. Targeting of cancer cells which express NTSR1 ectopically is a useful strategy for both detection and therapy of NTSR1 expressing cancers. The rapidly expanding fields of nanotherapeutics and immune therapeutics may be able to take advantage of NTS and NTSR to advance the treatment of CRC and other cancers. The technological aspects await validation in the clinical setting.

Antagonists of NTSRs, much like tamoxifen in breast cancer, may be a hitherto unexplored endocrine modulatory chemotherapy in patients with CRC expressing NTS/NTSR. The efficacy and safety of these antagonists require future clinical studies to assess both its safety and efficacy before it can be used in the treatment of cancers.

The road to developing a new cancer drug is long and arduous. For example, in the case of breast cancer, the role oestrogen played was described as early as 1896. George Beatson found that the lives of breast cancer patients were extended by bilateral oophorectomies. It took eight decades of work for tamoxifen, the oestrogen receptor antagonist, to be used in the treatment of breast cancer. Even more recently, almost 20 years separated the discovery of the HER2 receptor and the licensing of Herceptin (trastuzumab). It is clear that significant future work and investment is required if NTS pathways are to be exploited for the benefit of patients with cancer. The increasing body of evidence should propel ongoing research and be built upon by robust prospective studies to achieve this goal.
